# Amino-Acid-Balanced Low-Protein Diets Reduce Nitrogen Excretion Without Affecting Growth Performance in Broilers

**DOI:** 10.3390/ani16030494

**Published:** 2026-02-04

**Authors:** Fumika Nanto-Hara, Tomoka Ema, Haruhiko Ohtsu

**Affiliations:** Division of Meat Animal and Poultry Research, Institute of Livestock and Grassland Science, National Agriculture and Food Research Organization, Tsukuba 305-0901, Ibaraki, Japan; hara.fumika186@naro.go.jp (F.N.-H.); ema.tomoka885@naro.go.jp (T.E.)

**Keywords:** amino-acid-balanced low-protein diets, broiler, nitrogen excretion, performance

## Abstract

Reducing greenhouse gas emissions from the livestock industry is essential for climate change mitigation. In poultry production, nitrous oxide (N_2_O) emissions from composted manure are a major concern because they are closely associated with nitrogen content in the manure. This study investigated whether feeding broilers amino-acid-balanced low-crude-protein (AALP) diets throughout the entire rearing period could reduce nitrogen excretion without affecting growth performance. Thirty-six male broiler chicks were assigned to a control diet or an AALP diet, and nitrogen excretion was estimated over 50 days using chromic oxide as an indigestible marker. Overall growth performance was maintained in the AALP group, with a significant improvement in feed conversion ratio during the grower phase. Organ weights were largely unaffected, although liver weight tended to increase. Nitrogen excretion was significantly reduced at most time points in the AALP group, with a cumulative reduction of 31.1% compared to the control, as estimated by model-based integration over days 7–47. These findings suggest that our designed AALP diets can effectively reduce nitrogen excretion while maintaining productivity, thereby contributing to sustainable poultry production and supporting national climate goals.

## Simple Summary

Livestock farming produces greenhouse gases, including nitrous oxide (N_2_O), which is released from chicken manure during composting. Because N_2_O emissions are associated with nitrogen levels in manure, reducing nitrogen excretion is an important mitigation strategy. This study examined whether feeding broiler chickens a low-crude-protein diet, balanced with amino acids, could lower nitrogen excretion without compromising growth performance. Chickens were fed a control or an amino-acid-balanced low-protein (AALP) diet for 50 days. Birds receiving the AALP diet showed maintained growth, had improved feed efficiency during the grower phase, and excreted approximately 31% less nitrogen. These results suggest that AALP diets can reduce nitrogen output while maintaining productivity, thereby lowering the substrate for downstream emissions and offering a practical approach toward sustainable poultry farming.

## 1. Introduction

Addressing climate change is a global challenge, and reducing greenhouse gas emissions from agri-food systems is a critical component of national mitigation strategies. In the livestock sector, nitrous oxide (N_2_O) emissions arising from manure management are closely associated with dietary nitrogen intake and excretion.

Efficient nitrogen (N) management in broiler nutrition is central to both productivity and environmental stewardship. In fast-growing strains, amino acid (AA)-balanced low-crude-protein (AALP) diets—characterized by reduced crude protein (CP) supplemented with feed-grade indispensable (essential) amino acids (IAAs)—consistently lower N excretion with minimal growth penalties when energy density and ideal AA ratios are maintained. A recent meta-analysis in modern broilers indicates that each one-percentage-point reduction in CP reduces daily N excretion by 10% under AA-adequate conditions, while average daily gain and feed intake can be maintained; modest increases in feed conversion may occur depending on formulation and production phase [1].

Mechanistically, lowering dietary CP primarily reduces the intake of nonlimiting amino–nitrogen, thereby attenuating hepatic deamination and uric-acid-based N disposal, the dominant terminal pathway in birds [2,3]. When limiting IAAs are supplied in crystalline form and standardized ideal digestible ratios relative to lysine are maintained, near-complete digestibility and rapid absorption kinetics can meet postabsorptive demands despite reduced intact protein. Consequently, ammonia formation via deamination and downstream uric acid production decline, diminishing N substrates in manure that can be mineralized to ammonia and, through nitrification–denitrification, contribute to potential N_2_O formation—pathways explicitly considered in inventory guidance [4,5].

From an accounting perspective, the 2006 IPCC Guidelines and the 2019 Refinement explicitly link dietary N intake, N excretion, and manure management to national inventory estimates for livestock CH_4_/N_2_O, underscoring nutrition as a primary mitigation lever alongside housing and manure practices [5,6]. In Japan, the 2025 Plan for Global Warming Countermeasures sets strengthened interim targets (−46% by FY2030, −60% by FY2035, −73% by FY2040 vs. FY2013) toward net-zero emissions by 2050, further incentivizing nutrition-based measures that preserve productivity while reducing manure-related emissions [7,8].

Recent reviews emphasize that successful reduced-CP programs require phase-specific IAA profiling and careful attention to conditionally essential and nonessential AAs—notably glycine/serine equivalents—given the differences in digestive dynamics between protein-bound and crystalline AAs. These reviews also highlight systems-level co-benefits (e.g., reduced reliance on soybean meal and lower acidification and eutrophication potentials), while identifying knowledge gaps in immunity, welfare indices, sex-specific requirements, and economics. Such gaps motivate whole-cycle evaluations in contemporary high-growth genotypes [4,9].

Regarding the time window of intervention, not all studies are confined to a single phase: several investigations and syntheses have evaluated multi-phase or extended programs, and life-cycle assessments have modeled CP reductions across grower and finisher phases with demonstrable environmental gains [1]. For example, starter-phase AALP programs reduced N excretion, albeit with marginal performance penalties in some settings, whereas finisher-phase formulations (e.g., 19→17% CP at ideal AA ratios) decreased N output without growth impairment; the role of nonessential AA (glycine/serine equivalents) has also been highlighted [10,11,12,13]. Moreover, phase-targeted trials have shown that reduced-CP diets supplemented with essential AAs can lower nitrogen excretion without compromising productivity during the starter or finisher periods [6,7,8,9,10]. Nevertheless, comprehensive “entire rearing period” assessments—quantifying performance and N outcomes under consistent diets and analytics from placement to slaughter—remain relatively limited, supporting the need for integrated, full-cycle studies in modern fast-growing broilers [1,8].

Therefore, the objective of this study was to test an AA-adequate, reduced-CP (AALP) feeding strategy across the full rearing period, designed to lower N excretion without impairing productivity. We evaluated growth performance, organ traits, plasma biochemistry and AAs, and total N digestibility/excretion. To facilitate benchmarking against production practice, we additionally report the European Production Efficiency Factor (EPEF) and days to reach 2.25 kg. In defining CP targets, control diets were anchored to commonly used commercial feeds in Japan and reduced-CP levels were established from preliminary animal trials, identifying the lowest CP that maintained normal growth under our conditions; full analytical details are provided in the Materials and Methods Section. This work quantifies nutrition-based N mitigation consistent with international inventory guidance and national decarbonization goals [5,6,7,8].

## 2. Materials and Methods

### 2.1. Ethics Statement

All procedures were approved by the Animal Care Committee of the Institute of Livestock and Grassland Science, National Agriculture and Food Research Organization, Japan (Approval number: 21B177ILGS), and adhered to the ARRIVE guidelines. All experiments were performed in accordance with the relevant guidelines and regulations.

### 2.2. Animals and Experimental Design

Two dietary treatments were designed to evaluate the effect of reduced CP levels on nitrogen excretion in broilers. CP levels for each diet were determined based on two criteria: (i) the control diet reflected the CP content of commercially available broiler feeds commonly used in Japan; (ii) the minimum CP level for the low-CP diet was established through multiple preliminary animal trials, which demonstrated that 19% CP during the starter phase and 18% CP during the grower phase maintained normal growth performance. Accordingly, the control group was fed a conventional diet with a CP content of 22% during the starter phase and 20% during the grower phase, whereas the AALP group was fed an AALP diet with a CP content of 19% during the starter phase and 18% during the grower phase. Experimental feed compositions are detailed in Table 1. All diets were formulated mainly with corn and soybean meal, were isocaloric (13.0 MJ/kg), and were designed to provide all essential amino acids at levels that are at least 1.1-fold higher than the Japanese Feeding Standard requirements for poultry [14].

Newly hatched, day-old male Ross 308 broiler chicks (Komatsu Hatchery, Nagano, Japan) were reared for 50 days under controlled environmental conditions. At hatching, birds received standard vaccinations, including a Marek’s disease bivalent live vaccine (HVT + SB-1, Kyoritsu Seiyaku Corporation, Tokyo, Japan) and fowl pox live vaccine (ChickNPOX, VAXXINOVA, Tokyo, Japan). Chicks were randomly allocated to dietary treatments at the pen level, which served as the experimental unit for all analyses; randomization was not stratified by body weight. During the starter phase (d 1–21), birds were housed at three birds per pen in wire cages (42 × 34 cm) to facilitate close monitoring of health status and early feed intake. During the grower phase (d 22–50), birds remained at three birds per pen in larger floor pens (84 × 52 cm) bedded with wood shavings. For individual fecal collection during the grower period, two birds per pen were temporarily transferred to individual cages (84 × 26 cm; one bird/cage) for two days to allow accurate total excreta collection. Consequently, during the grower phase, the number of replicates per treatment was reduced (from six to four pens; three birds per pen) to ensure adequate space allowance per bird in accordance with animal-welfare standards while maintaining accurate measurements of growth performance and nitrogen excretion.

Temperature was maintained at 32–34 °C during the first week and then reduced by approximately 3 °C per week to 24 °C. Relative humidity was maintained at 50–60% throughout the experiment. A daily dark period of 4 h was provided in accordance with animal welfare recommendations. Ventilation was provided via a standard HVAC system typical of research facilities. Feed and water were offered ad libitum using bucket-type feeders and drinkers (not nipple systems). These housing and management conditions align with current broiler welfare guidelines.

To estimate nitrogen excretion, 0.1% chromic oxide was added to the diets as an indigestible marker. Fecal samples were collected at seven time points: days 6–8, 13–15, 18–20, 27–29, 34–36, 41–43, and 46–48. During the grower phase, two birds from each pen were transferred to individual cages for 2 days to allow accurate fecal collection. Samples were dried in a forced oven at 55 °C for 60 h and stored at room temperature until further use.

Body weight (BW) and feed intake (FI) of the birds were recorded once a week and, on the final day of the experimental period, two birds per pen were sampled and euthanized via rapid decapitation followed by exsanguination using appropriate equipment, in accordance with the American Veterinary Medical Association Guidelines for the Euthanasia of Animals (2020 edition) [15]. The pectoralis major muscle, liver, abdominal fat, and spleen were excised and weighed immediately. The EPEF and the estimated number of days required to reach a slaughter weight of 2.25 kg were calculated at the pen level. EPEF was computed as follows:

$$EPEF=\;\left(\frac{Livability\;\%\;\times Final\;BW\;kg}{FCR}\right)\times \left(\frac{100}{Age\;d}\right)$$

The number days to 2.25 kg was estimated by linear interpolation between successive weekly body weight measurements.

### 2.3. Dietary AA Analysis

AAs in diets were quantified using Shokukanken Inc. (Maebashi, Japan) in accordance with the Standard Tables of Food Composition in Japan—Analytical Manual (2015, 7th rev. ed.), Chapter 4 [16]. Samples were ground and sieved (1 mm). General AAs were determined after sealed-tube acid hydrolysis (6 mol/L HCl, 110 °C, 24 h) using ion-exchange chromatography with post-column derivatization [16,17]. The suitability of these hydrolysis conditions is supported by previous evaluations of time, temperature, and antioxidant effects in protein hydrolysis [18].

Sulfur-containing AAs were oxidized with performic acid before hydrolysis and quantified chromatographically. Tryptophan was analyzed using reverse-phase HPLC following alkaline hydrolysis, as recommended for acid-labile residues. These procedures follow internationally recognized protocols, including the AOAC Official Methods for AA profiling and tryptophan analysis [17].

### 2.4. Nitrogen Excretion Measurement

Diets and dried fecal samples were milled using a Retsch ZM 100 ring sieve mill fitted with a 1 mm screen (Retsch GmbH & Co., Haan, Germany). Total nitrogen was determined by the combustion (Dumas) method using a Sumigraph NC-TRINITY nitrogen analyzer (Sumika Chemical Analysis Service, Tokyo, Japan), and CP was calculated as CP = N × 6.25. The Dumas procedure conformed to the AOAC Official Method (OMA, 22nd ed.) [17].

Chromic oxide concentrations in diets and feces were determined using the wet acid digestion method described by Bolin et al. [19], and nutrient analyses were performed according to the procedures outlined by the Association of Official Analytical Chemists [17]. Total fecal volume was calculated from the amount of chromium oxide ingested and recovered in feces, assuming a recovery rate of 100%. Nitrogen digestibility and excretion were calculated using the chromic oxide indicator method as follows (all concentrations are expressed on a dry-matter basis):

$$Apparent\;N\;digestibility=1-\left(\frac{{Cr}_{feces}}{{Cr}_{diet}}\right)\times \left(\frac{N_{diet}}{N_{feces}}\right)$$

$$Fecal\;DMoutput\left(\frac{g}{d}\right)=\frac{{Cr}_{intake}\left(\frac{g}{d}\right)}{{Cr}_{feces}}=\frac{{FI}_{DM}\times {Cr}_{diet}}{{Cr}_{feces}}$$

$$Fecal\;N\;excretion\;\left(\frac{g}{d}\right)=Fecal\;DMoutput\;\left(\frac{g}{d}\right)\times N_{feces}$$

To estimate cumulative nitrogen excretion over the whole rearing period, daily nitrogen excretion values measured on days 7, 14, 19, 28, 35, 42, and 47 were fitted using a cubic regression model for each dietary group. The definite integral of each fitted regression equation over the interval from day 7 to day 47 was then calculated. This integration-based approach quantified the area under the regression curve, providing an estimate of total nitrogen excretion across the experimental period.

### 2.5. Blood Collection and Analysis

On the final day of the experimental period, blood samples were collected by venipuncture from the branchial vein of two birds per pen. Blood samples were centrifuged at 3000× *g* for 15 min at 10 °C to separate plasma which was then transferred into 1.5 mL labeled vials and stored at −20 °C until further use. Plasma concentrations of total protein, total cholesterol, triglycerides, phospholipids, HDL-cholesterol, LDL-cholesterol, as well as the activities of AST and ALT were determined by Kotobiken Medical Laboratories (Ibaraki, Japan).

### 2.6. Plasma AA Analysis

Plasma-free AAs were quantified by NDTS Co., Ltd. (Sapporo, Japan) using liquid chromatography–tandem mass spectrometry (LC–MS/MS). Plasma samples were deproteinized by acid treatment and derivatized with propyl chloroformate, a widely used chloroformate-based reagent for AA derivatization in physiological samples [20]. After derivatization, samples were subjected to LC–MS/MS for the quantification of 20 AAs.

The analytical system comprised an HPLC unit (Prominence, Shimadzu Corp., Kyoto, Japan) coupled to a tandem mass spectrometer (MS-8040, Shimadzu Corp.). Detection and quantification were performed in multiple reaction monitoring mode, following established LC–MS/MS practices for plasma AA profiling [21] and validated workflows for Shimadzu triple-quadrupole systems [22]. Calibration curves were generated using authentic standards for each AA, and sample concentrations were calculated from peak areas relative to the corresponding standard curves. All measurements followed the manufacturer’s guidelines and established LC–MS/MS best-practice procedures.

### 2.7. Statistical Analysis

Statistical analyses were performed in R (version 4.3.3) using the lme4 (v1.1-38), lmerTest (v3.2-0), and emmeans (v2.0.1) packages, with statistical significance declared at *p* < 0.05. BW, BWG, FI, and FCR were analyzed using linear mixed-effects models including fixed effects of diet, period (Starter vs. Grower), their interaction, and a random intercept for pen (experimental unit). FCR was log-transformed prior to analysis to improve model assumptions, and estimated marginal means (EMMs) with 95% confidence intervals were back-transformed to report geometric means. *p* values and denominator degrees of freedom were obtained using the Satterthwaite approximation via lmerTest. Pairwise comparisons between diets were performed within each period based on EMMs, using Tukey adjustment for multiple comparisons by default. All inferences were made at the pen level.

Performance indices that were not repeatedly measured (EPEF and days to reach 2.25 kg) were compared between diets using two-sample *t*-tests at the pen level.

Weekly repeated measurements of fecal nitrogen excretion and nitrogen digestibility at seven time points (6–8, 13–15, 18–20, 27–29, 34–36, 41–43, and 46–48 d) were analyzed using linear mixed-effects models with fixed effects of diet, period (7 levels), and their interaction, and a random intercept for pen. Models were fitted by REML; when singular fits were detected, results were interpreted with caution, but the specified random-effects structure was retained. For nitrogen outcomes, EMMs with 95% confidence intervals were reported, and period-specific pairwise comparisons between diets were conducted with Tukey adjustment.

For terminal measurements (organ weights, plasma AA profiles, and plasma clinical biochemistry), values from two birds per pen were averaged to obtain pen-level means, which were compared between diets using two-sided *t* tests (Welch’s correction applied when variances were unequal). Results are presented as mean ± SEM.

## 3. Results

BW did not differ significantly between the CONT and AALP groups in either the starter or grower period (Table 2). EMMs for BW were 810.96 g (95% CI: 605.83–1016.09 g) in CONT and 810.88 g (605.75–1016.01 g) in AALP during the starter period (*p* = 1.00), and 2999.46 g (2739.98–3258.93 g) and 3310.25 g (3050.78–3569.72 g), respectively, during the grower period (*p* = 0.09). Similarly, BWG was not significantly affected by diet in either period. During the starter period, BWG averaged 36.54 g (29.36–43.71) in CONT and 36.53 g (29.35–43.70) in AALP (*p* = 1.00). During the grower period, BWG tended to be higher in the AALP group (89.66 g; 80.58–98.73) than in the CONT group (78.27 g; 69.19–87.34), although the difference did not reach statistical significance (*p* = 0.077).

FI was similar between dietary treatments throughout the experimental period. In the starter phase, FI was 50.04 g (42.73–57.34 g) in CONT and 52.34 g (45.03–59.64 g) in AALP (*p* = 0.65), and during the grower phase it was 137.31 g (128.07–146.55 g) and 145.56 g (136.32–154.80 g), respectively (*p* = 0.19). FCR, analyzed on the log scale and reported as back-transformed geometric means, did not differ between treatments during the starter period (0.263 [0.213–0.313] for CONT vs. 0.308 [0.258–0.358] for AALP; *p* = 0.20). In contrast, during the grower period, AALP birds exhibited a significantly improved FCR compared with CONT birds (0.509 [0.446–0.572] vs. 0.609 [0.546–0.672], respectively; *p* = 0.031).

Non-repeated performance indicators (EPEF, and days to 2.25 kg) exhibited no significant differences between dietary treatments (Table 3). EPEF tended to be higher and the number of days required to reach 2.25 kg tended to be fewer in the AALP group; however, these differences were not statistically significant. Chicks in the AALP group exhibited an increasing trend in liver weight (*p* = 0.08), but the two groups did not differ significantly in the weights of the pectoralis major muscle, abdominal fat, and spleen.

Nitrogen excretion was markedly reduced in the AALP group compared with the CONT group across most of the experimental period (Table 4). From days 13–15 onward, nitrogen excretion was significantly lower in the AALP group, including on days 13–15 (0.428 vs. 0.641 g/day; *p* = 0.010), days 18–20 (0.567 vs. 0.832 g/day; *p* < 0.01), days 34–36 (0.809 vs. 1.139 g/day; *p* < 0.01), days 41–43 (0.768 vs. 1.321 g/day; *p* < 0.0001), and days 46–48 (0.979 vs. 1.350 g/day; *p* < 0.01). On days 27–29, nitrogen excretion tended to be lower in AALP than in CONT (0.658 vs. 0.847 g/day), although this difference was not significant (*p* = 0.059). No significant difference was observed on days 6–8 (*p* = 0.16).

Nitrogen digestibility was significantly higher in the AALP group during the early-growth stages. Specifically, digestibility increased on days 6–8 (96.43% vs. 95.24%; *p* < 0.01), days 13–15 (96.31% vs. 95.19%; *p* < 0.01), and days 18–20 (97.05% vs. 95.77%; *p* < 0.01). Digestibility tended to be higher in the AALP group on days 27–29 (*p* = 0.093) and days 34–36 (*p* < 0.01), whereas no significant differences were detected between treatments at days 41–43 (*p* = 0.14) or days 46–48 (*p* = 0.078).

**Figure 1:**
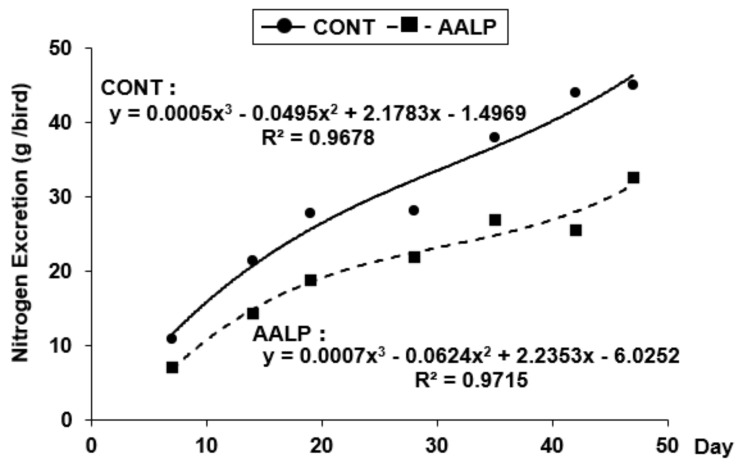
Estimated nitrogen excretion in broilers from days 7 to 47 based on cubic regression analysis. The estimated daily nitrogen excretion from days 7 to 47 based on quadratic regression using measured values at days 7, 14, 19, 28, 35, 42, and 47. CONT: control diet (CP 22% starter, 20% grower); AALP: AA-balanced low-protein diet (CP 19% starter, 18% grower). Nitrogen excretion was measured using the chromic oxide index method.

Figure 1 shows the estimated daily nitrogen excretion from days 7 to 47, calculated on the basis of cubic regression analysis conducted on the measured nitrogen excretion values on days 7, 14, 19, 28, 35, 42, and 47 (Table 4). The regression equations for the control and AALP groups were y = 0.0005x^3^ − 0.0495x^2^ + 2.1783x − 1.4969 (R^2^ = 0.9678) and y = 0.0007x^3^ − 0.0624x^2^ + 2.2353x − 6.0252 (R^2^ = 0.9715), respectively. To estimate the total nitrogen excretion over the rearing period, each regression equation was integrated from days 7 to 47. The resulting cumulative nitrogen excretion values for the control and AALP groups were 1257.4 and 866.3 g/bird, respectively. These findings indicate that administering an AALP diet throughout the rearing period can reduce nitrogen excretion by approximately 31.1% compared with conventional diets.

Plasma AA concentrations are shown in Table 5. Among essential AAs, concentrations of valine and histidine were significantly lower in the AALP group than the control group. Leucine and tryptophan concentrations tended to be lower (*p* = 0.06 and *p* = 0.05, respectively), whereas methionine tended to be higher (*p* = 0.05) in the AALP group than in control. No significant differences were observed between treatments for arginine, isoleucine, lysine, phenylalanine, or threonine. For nonessential AAs, no significant treatment effects were detected for alanine, asparagine, aspartic acid, glutamine, glutamic acid, serine, glycine, cystine, proline, or tyrosine. When AAs were grouped, the concentration of branched-chain AAs (leucine + isoleucine + valine) was significantly lower in the AALP group than in the control group, whereas total aromatic AAs (phenylalanine + tyrosine) did not differ significantly between treatments.

Blood biochemical parameters are summarized in Table 6. Triglyceride concentrations were significantly higher in the AALP group than in the control group. Total cholesterol tended to be higher in the AALP group (*p* = 0.08). However, no significant differences were observed in total protein, phospholipids, HDL-cholesterol, or LDL-cholesterol. Among liver-related enzymes, ALT activity tended to be higher in the AALP group than in the control (*p* = 0.05), whereas AST activity did not differ significantly between treatments. The atherosclerosis index was significantly higher in the AALP group than in the control group.

## 4. Discussion

In this study, we evaluated the effects of administering our designed AALP diets throughout the entire broiler rearing period on growth performance and nitrogen excretion in broilers. Our findings provide key insights into the development of sustainable poultry production strategies.

During the starter period, BW and BWG did not differ between treatments (*p* = 1.00), indicating that any early-life sensitivity to AA margins did not translate into detectable performance differences by day 21. In line with the analyzed AA contents shown in Table 1, the sum of glycine and serine (Gly + Ser) in the AALP diet was 18% lower than in the control during the starter period and 12% lower during the grower period, while still meeting the Japanese Feeding Standard [14]. This narrower margin could theoretically increase early sensitivity [12,23]; however, plasma-nonessential AAs did not differ between treatments, and no significant growth differences were observed by the end of the starter phase.

From the grower phase onward, BW tended to be higher in the AALP group (*p* = 0.09) and FCR was significantly improved during the grower period. This pattern is consistent with a possible metabolic adaptation—namely, reduced deamination of surplus AAs and lower nitrogen-disposal costs under a more targeted AA supply—as summarized in reviews of reduced-CP diets that emphasize diminished deamination when AA balance is optimized [2,3,4]. This interpretation is further supported by quantitative estimates of the high energetic demand associated with uric-acid-based nitrogen excretion in birds [24], together with aggregated and individual trial evidence showing that AA-adequate reduced-CP regimens can maintain or improve efficiency while lowering nitrogen excretion [1,25]. Transcriptomic analysis by Asiamah et al. [26] reported upregulation of genes related to protein metabolism and peroxisome proliferator-activated receptor signaling in the liver of broilers fed low-CP diets, which is consistent with improved nutrient utilization with or without additional supplementation of nonessential AAs. In terms of organ development, the AALP group exhibited a trend toward increased liver weight (*p* = 0.08), which may reflect hepatic adaptation; however, given only a borderline increase in ALT activity and unchanged AST, targeted hepatic assessments are required to clarify its biological significance. No significant differences were observed in the weights of the pectoralis major muscle, abdominal fat, or spleen, suggesting that the AALP diet had minimal impact on productivity and immune organ development. Consistent with the performance outcomes, non-repeated indicators (EPEF and days to reach 2.25 kg) did not differ significantly between treatments.

Plasma AAs indicated a tighter margin in the branched-chain AAs (BCAAs) under AALP (lower composite BCAA concentrations with select essential AAs trending downward), whereas nonessential AAs were broadly unchanged. Together with maintained BW/BWG and improved grower-phase FCR, these data suggest either narrower BCAA supply margins or enhanced utilization under AALP. This is consistent with experimental evidence, showing that optimization of BCAA ratios in reduced-CP diets sustains feed efficiency across growth phases [2,4,27,28]. Accordingly, further formulation refinements such as adjusting leucine-relative balance through adequate valine and isoleucine supply may help preserve metabolic buffering while the nitrogen-mitigation benefits of AALP strategies.

Blood biochemistry suggested a lipid metabolism shift under AALP, characterized by higher triglyceride concentrations, a higher atherogenic index, and a trend toward higher total cholesterol, whereas ALT showed only a borderline rise and AST/total protein remained unchanged. In birds, hepatic lipid handling relies primarily on liver-derived VLDL; thus, this profile is compatible with functional adaptation rather than overt hepatocellular injury, although targeted follow-up assessments (e.g., VLDL secretion, hepatic lipid content, and bile acids metabolisms) remain warranted [29,30,31]. The lack of an AST increase and stable total protein further argue against hepatocellular damage under avian clinical pathology criteria, supporting the interpretation that the increased liver weight reflects adaptive lipid handling rather than pathological enlargement [32].

From days 13–15 onward, nitrogen excretion was significantly lower in the AALP group at most time points (with no difference on days 6–8 and only a trend on days 27–29), with an estimated cumulative reduction of approximately 31.1% compared with the control group. This aligns with the findings of Belloir et al. [11] and Askri et al. [33], who demonstrated that optimizing AA balance improves nitrogen utilization efficiency and reduces environmental burden. Notably, the high coefficient of determination (R^2^ > 0.96) for the nitrogen-excretion regression models suggests good in-sample fit; however, external validation is required before using these models for prediction in feed design or environmental assessments. Although lower N excretion should reduce the substrate available for NH_3_/N_2_O formation during manure management, the N → N_2_O proportionality is system-dependent [34,35]. Therefore, converting the ~31.1% N reduction (days 7–47, model based) into N_2_O mitigation requires process-based modeling or direct flux measurements [36]. In this context, national-scale reviews of manure treatment in Japan emphasize that N_2_O formation during storage/composting is highly management-dependent, reinforcing the need for system-specific assessment [37]. At the system scale, life-cycle assessments also indicate that reduced-CP diets mainly express climate and air-quality benefits via the manure-management module, with magnitude contingent on local practices [38].

While several studies have examined reduced-CP strategies within specific phases, extended and full-period programs have also been reported [1]. Building on the literature, this study evaluated the effects of the continuous AALP program throughout the entire rearing period. Importantly, the study used modern, fast-growing broiler strains that have been genetically improved for growth performance and feed efficiency, reflecting current commercial production. To enhance practical relevance, we also reported production-oriented endpoints (e.g., EPEF and days to reach market weight) and interpreted nitrogen outcomes within inventory-consistent frameworks. Additionally, nitrogen excretion was measured at seven different time points, allowing for a time-course analysis and model-based integration to estimate cumulative nitrogen output over days 7–47, rather than relying on single-point measurements. Together, these features highlight the novelty of this study and provide valuable insights for future feed formulation and broiler management strategies aimed at improving nitrogen efficiency while alleviating environmental impact.

Overall, under diets anchored to commercial controls, the AALP strategy maintained growth performance, improved FCR during the grower phase, and substantially lowered cumulative nitrogen excretion, while revealing metabolic shifts (lipids and borderline ALT) that warrant targeted hepatic assessments. Future work should validate these findings across genetic strains, stocking densities, and formulations, and quantify downstream emissions within standard inventory frameworks.

Several limitations should be considered when interpreting these findings. The study lacked full nutrient composition profiling, measurements of ammonia N or uric acid, and sufficiently detailed ingredient cost data to support a formal economic analysis. Meat quality endpoints and histological assessments, including liver histology, were not evaluated. In addition, the number of pens was modest, and direct N_2_O emissions from manure were not measured. These constraints limit mechanistic interpretation, economic evaluation, and environmental inference. Nevertheless, the time course nitrogen data and performance outcomes provide practical guidance for future feed formulation strategies, and support demonstration-scale studies incorporating greater replication, comprehensive nutrient analyses, economic evaluation, and process-level emission measurements.

## 5. Conclusions

The administration of our designed AALP diets throughout the entire broiler rearing period substantially reduced nitrogen excretion (~31% cumulative reduction estimated by model-based integration over days 7–47). Overall growth performance was maintained, with a significant improvement in feed efficiency during the grower phase, consistent with a physiological adaptation to the reduced-CP, AA-adequate formulation. These results support the development of sustainable poultry production systems and align with national climate goals by providing inventory-relevant evidence for nutrition-based nitrogen mitigation. In parallel, blood biochemistry and organ data indicated shift in a lipid metabolism without evidence of overt hepatocellular injury, supporting AALP as a practical and scalable strategy to reduce environmental impact. Future work should extend these findings to quantify downstream emissions within standard frameworks.

## Figures and Tables

**Table 1:** Ingredients and chemical composition of experimental diets.

Ingredients, %	Starter Period, 0–3 Weeks		Grower Period, 4–7 Weeks	
	CONT	AALP	CONT	AALP
Corn	54.147	65.315	60.715	67.681
Soybean meal	37.420	26.655	32.517	25.914
Vegetable oil	4.471	2.759	3.533	2.513
Calcium carbonate	1.085	1.079	0.990	0.986
Dibasic calcium phosphate hydrate	1.551	1.701	1.329	1.421
Sodium chloride	0.466	0.467	0.340	0.340
DL-methionine	0.331	0.430	0.151	0.211
Lys-Cl	0.098	0.449	-	0.208
Arg	-	0.234	-	0.096
Trp	-	0.029	-	-
Ileu	-	0.145	-	0.045
Thr	0.024	0.189	-	0.119
Val	-	0.141	-	0.060
Vitamin mixture ^1^	0.200	0.200	0.200	0.200
Mineral mixture ^2^	0.100	0.100	0.100	0.100
Selenium	0.006	0.006	0.006	0.006
Calculated value				
Metabolizable energy ^3^, MJ/kg	13.0	13.0	13.0	13.0
Crude protein, %	22.00	19.00	20.00	18.00
Analytical value, %				
Arginine	1.40	1.30	1.20	1.10
Isoleucine	0.94	0.90	0.87	0.79
Leucine	1.70	1.50	1.70	1.50
Lysine	1.20	1.20	1.00	1.10
Methionine	0.38	0.34	0.30	0.29
Phenylalanine	1.10	0.86	0.98	0.87
Threonine	0.77	0.77	0.71	0.71
Tryptophan	0.26	0.23	0.49	0.21
Valine	1.00	0.97	0.95	0.90
Histidine	0.65	0.54	0.56	0.53
Alanine	1.10	0.90	1.00	0.91
Aspartic acid	2.20	1.80	2.00	1.70
Glutamic acid	3.80	3.10	3.50	3.10
Serine	0.98	0.80	0.92	0.81
Glycine	0.90	0.74	0.84	0.74
Cystine	0.34	0.26	0.30	0.28
Proline	1.20	1.00	1.10	1.10
Tyrosine	0.61	0.51	0.55	0.50
Crude protein	22.2	19.0	20.3	18.1

^1^ Vitamin mixture provided the following (per kilogram of diet): vitamin A (from retinyl acetate), 4000 IU; cholecalciferol, 600 IU; vitamin E (from DL-α-tocopheryl acetate), 15 IU; vitamin K (menadione sodium bisulfate), 1.5 mg; riboflavin, 10 mg; D-calcium pantothenate, 20 mg; nicotinic acid, 50 mg; choline chloride, 500 mg; pyridoxine hydrochloride, 3 mg; folic acid, 2 mg; thiamine mononitrate, 3 mg; D-biotin, 0.3 mg; vitamin B12 (cyanocobalamin), 20 μg. ^2^ Mineral mixture provided the following (per kilogram of diet): iron (FeSO_4_·7H_2_O), 80 mg; manganese (MnCO_3_·nH_2_O), 60 mg; zinc (ZnO), 40 mg; copper (CuSO_4_·5H_2_O), 8 mg; iodine (calcium iodate), 0.5 mg. ^3^ Metabolizable energy (ME) values were estimated based on ingredient tables in the Japanese Feeding Standard for poultry, and all experimental diets were formulated to be isocaloric on an ME basis. CONT: control diet; AALP: low-crude-protein diet supplemented with amino acids.

**Table 2:** Growth performance by diet and period (EMMs with 95% CIs); within-period contrasts between CONT and AALP.

	CONT (EMM, 95% CI)	AALP (EMM, 95% CI)	Δ (AALP−CONT)	*p*-Value
Body weight, g				
21 d	810.96 (605.83–1016.09)	810.88 (605.75–1016.01)	−0.08	1.00
50 d	2999.46 (2739.98–3258.93)	3310.25 (3050.78–3569.72)	310.79	0.09
Body weight gain, g/d				
Starter period	36.54 (29.36–43.71)	36.53 (29.35–43.70)	−0.01	1.00
Grower period	78.27 (69.19–87.34)	89.66 (80.58–98.73)	11.39	0.08
Feed intake, g/day				
Starter period	50.04 (42.73–57.34)	52.34 (45.03–59.64)	2.30	0.65
Grower period	137.31 (128.07–146.55)	145.56 (136.32–154.80)	8.26	0.19
Feed conversion rate, g:g				
Starter period	0.263 (0.213–0.313)	0.308 (0.258–0.358)	0.05	0.20
Grower period	0.609 (0.546–0.672)	0.509 (0.446–0.572)	−0.100	0.03

Values are EMMs with 95% CIs at the pen level (experimental unit). Δ (AALP−CONT) and *p* values are from within-period contrasts of EMMs under a linear mixed-effects model (fixed effects: diet, period [Starter vs. Grower], diet × period; random intercept: pen; Satterthwaite df). FCR was analyzed on the log scale and reported as back-transformed geometric means; contrasts were tested on the log scale. Starter: 3 birds/pen, *n* = 6 pens/treatment; Grower: 3 birds/pen, *n* = 4 pens/treatment. Significance was declared at *p* < 0.05; trend was defined as 0.05 ≤ *p* < 0.10. Initial BW did not differ between treatments and averaged 43.8 ± 1.4 g (mean ± SEM). CONT: control diet (CP 22% starter, 20% grower); AALP: AA-balanced low-protein diet (CP 19% starter, 18% grower).

**Table 3:** Performance index and organ weights of broilers fed with CONT and AALP on day 50.

	CONT	AALP	*p*-Value
Performance index			
EPEF, points	358.42 ± 6.91	414.07 ± 48.47	0.30
Days to 2.25 kg, d	42.91 ± 0.39	40.91 ± 1.69	0.29
Organ weight, g			
*pectoralis major*	304.4 ± 79.4	335.8 ± 57.5	0.38
Liver	48.2 ± 12.7	60.0 ± 12.1	0.08
Abdominal fat	18.6 ± 12.0	29.1 ± 13.9	0.13
Spleen	3.7 ± 1.0	4.3 ± 1.5	0.33

Values are mean ± SEM at the pen level. Starter phase: three birds per pen, *n* = 6 pens per treatment; Grower phase: three birds per pen, *n* = 4 pens per treatment. CONT: control diet (CP 22% starter, 20% grower); AALP: AA-balanced low-protein diet (CP 19% starter, 18% grower).

**Table 4:** Period-specific EMMs (95% CIs) for nitrogen excretion (g/day) and nitrogen digestibility (%), with within-period contrasts between CONT and AALP across seven time points (6–8 to 46–48 d).

	CONT (EMM, 95% CI)	AALP (EMM, 95% CI)	Δ (AALP−CONT)	*p*
Nitrogen excretion, g/day				
6–8 d	0.326 (0.196–0.455)	0.212 (0.082–0.341)	−0.114	0.159
13–15 d	0.641 (0.512–0.770)	0.428 (0.299–0.557)	−0.213	0.01
18–20 d	0.832 (0.702–0.961)	0.567 (0.438–0.696)	−0.265	<0.01
27–29 d	0.847 (0.692–1.002)	0.658 (0.503–0.813)	−0.189	0.06
34–36 d	1.139 (0.984–1.293)	0.809 (0.655–0.964)	−0.329	<0.01
41–43 d	1.321 (1.167–1.476)	0.768 (0.614–0.923)	−0.553	<0.0001
46–48 d	1.350 (1.196–1.505)	0.979 (0.825–1.134)	−0.371	<0.01
Nitrogen digestibility, %				
6–8 d	95.24 (94.68–95.81)	96.43 (95.86–96.99)	1.19	<0.01
13–15 d	95.19 (94.62–95.76)	96.31 (95.74–96.87)	1.12	<0.01
18–20 d	95.77 (95.21–96.34)	97.05 (96.48–97.61)	1.27	<0.01
27–29 d	95.08 (94.39–95.77)	95.85 (95.16–96.54)	0.78	0.09
34–36 d	94.69 (94.00–95.38)	96.07 (95.38–96.76)	1.38	<0.01
41–43 d	95.30 (94.61–95.99)	95.99 (95.30–96.68)	0.69	0.14
46–48 d	95.67 (94.98–96.36)	96.49 (95.80–97.18)	0.82	0.08

Values are EMMs (95% CIs) at the pen level. Δ (AALP−CONT) and *p* values are from period-specific contrasts under a mixed-effects model (fixed effects: diet, period [7 levels: 6–8, 13–15, 18–20, 27–29, 34–36, 41–43, 46–48 d], diet × period; random intercept: pen; Satterthwaite df). Significance: *p* < 0.05; trend: 0.05 ≤ *p* < 0.10. CONT: control diet (CP 22% starter, 20% grower); AALP: AA-balanced low-protein diet (CP 19% starter, 18% grower). Nitrogen excretion was measured using the chromic oxide index method.

**Table 5:** Plasma AA profile of broilers fed with CONT and AALP.

	CONT	AALP	*p*-Value
Amino acid concentration, nmol/mL			
Arginine	724.7 ± 29.1	805.6 ± 36.7	0.14
Isoleucine	100.0 ± 0.4	93.6 ± 2.1	0.16
Leucine	223.3 ± 5.0	209.9 ± 3.1	0.06
Lysine	101.8 ± 15.7	96.8 ± 3.4	0.77
Methionine	54.5 ± 2.3	62.7 ± 2.5	0.05
Phenylalanine	159.1 ± 2.4	149.7 ± 6.7	0.24
Threonine	416.4 ± 28.6	428.5 ± 44.1	0.83
Tryptophan	106.3 ± 3.7	96.6 ± 1.7	0.05
Valine	204.0 ± 5.1	184.5 ± 2.0	0.01
Histidine	96.5 ± 6.9	71.1 ± 3.6	0.02
Alanine	850 ± 37.8	844.9 ± 44.8	0.93
Asparagine	128.1 ± 8.3	137.3 ± 11.3	0.54
Aspartic acid	70.0 ± 10.6	63.1 ± 7.6	0.61
Glutamine	1274.8 ± 37.6	1220.6 ± 43.4	0.38
Glutamic acid	225.6 ± 12.4	202.5 ± 4.9	0.13
Serine	488.8 ± 38.3	519.4 ± 25.4	0.53
Glycine	549.9 ± 30.0	589.9 ± 39.1	0.45
Cystine	33.7 ± 2.8	34.3 ± 3.9	0.9
Proline	336.4 ± 10.6	363.4 ± 26.3	0.38
Tyrosine	301.7 ± 22.8	276.3 ± 20.6	0.44
Aromatic amino acids (Phe + Tyr)	460.8 ± 25.1	426.0 ± 21.9	0.34
Branched-chain amino acid (Leu + Ile + Val)	527.2 ± 11.8	487.9 ± 6.1	0.02

Values are presented as mean ± SEM; experimental unit = pen. three birds per pen, *n* = 4 pens per treatment. CONT: control diet (CP 22% starter, 20% grower); AALP: AA-balanced low-protein diet (CP 19% starter, 18% grower).

**Table 6:** Plasma biochemistry profile of broilers fed with CONT and AALP.

	CONT	AALP	*p*-Value
Total protein, mg/dL	3.0 ± 0.1	3.3 ± 0.1	0.22
Total cholesterol, mg/dL	118.3 ± 4.9	129.1 ± 1.4	0.08
Tryglycerides, mg/dL	30.5 ± 2.4	40.9 ± 3.1	0.04
Phospholipids, mg/dL	206.3 ± 9.0	230.0 ± 3.3	0.73
HDL-cholesterol, mg/dL	81.6 ± 2.9	83.6 ± 1.1	0.54
LDL-cholesterol, mg/dL	21.5 ± 1.2	24.4 ± 1.0	0.12
AST, units/L	420.8 ± 21.4	523.4 ± 71.5	0.22
ALT, units/L	1.0 ± 0.0	1.5 ± 0.2	0.05
Atherosclerosis index	0.45 ± 0.02	0.56 ± 0.04	0.04

Values are presented as mean ± SEM; experimental unit = pen. Three birds per pen, *n* = 4 pens per treatment. CONT: control diet (CP 22% starter, 20% grower); AALP: AA-balanced low-protein diet (CP 19% starter, 18% grower).

## Data Availability

The datasets generated and/or analyzed in the current study are available from the corresponding author on reasonable request.

## Abbreviations

The following abbreviations are used in this manuscript:

AALP	amino-acid-balanced low-crude-protein
CONT	control
CP	crude protein
N_2_O	nitrous oxide

## References

[1] Rauglaudre T.D., Méda B., Fontaine S., Lambert W., Fournel S., Létourneau-Montminy M.-P. (2023). Meta-Analysis of the Effect of Low-Protein Diets on the Growth Performance, Nitrogen Excretion, and Fat Deposition in Broilers. Front. Anim. Sci..

[2] Liu S.Y., Macelline S.P., Chrystal P.V., Selle P.H. (2021). Progress towards Reduced-Crude Protein Diets for Broiler Chickens and Sustainable Chicken-Meat Production. J. Anim. Sci. Biotechnol..

[3] Selle P.H., Macelline S.P., Chrystal P.V., Liu S.Y. (2023). The Challenge to Reduce Crude Protein Contents of Wheat-Based Broiler Diets. Anim. Prod. Sci..

[4] Selle P.H., Macelline S.P., Chrystal P.V., Liu S.Y. (2023). A Reappraisal of Amino Acids in Broiler Chicken Nutrition. World’s Poult. Sci. J..

[5] IPCC (2006). Emissions from Livestock and Manure Management. 2006 IPCC Guidelines for National Greenhouse Gas Inventories.

[6] IPCC (2019). 2019 N_2_O Emissions from Managed Soils. Refinement to the 2006 IPCC Guidelines for National Greenhouse Gas Inventories.

[7] Ministry of the Environment, Japan (MOEJ) (2025). Plan for Global Warming Countermeasures (Cabinet Decision, 18 February 2025; English Materials).

[8] Ministry of the Environment, Japan (MOEJ) (2025). Progress of the Plan for Global Warming Countermeasures in FY2023.

[9] Salahi A., Shahir M.H., Attia Y.A., Fahmy K.N.E., Bovera F., Tufarelli V. (2025). Impact of Low-Protein Diets on Broiler Nutrition, Production Sustainability, Gene Expression, Meat Quality and Greenhouse Gas Emissions. J. Appl. Anim. Res..

[10] Bregendahl K., Sell J., Zimmerman D. (2002). Effect of Low-Protein Diets on Growth Performance and Body Composition of Broiler Chicks. Poult. Sci..

[11] Belloir P., Méda B., Lambert W., Corrent E., Juin H., Lessire M., Tesseraud S. (2017). Reducing the CP Content in Broiler Feeds: Impact on Animal Performance, Meat Quality and Nitrogen Utilization. Animal.

[12] Dean D.W., Bidner T.D., Southern L.L. (2006). Glycine Supplementation to Low Protein, Amino Acid-Supplemented Diets Supports Optimal Performance of Broiler Chicks. Poult. Sci..

[13] El-far A.S., Kamiya M., Saneyasu T., Honda K. (2024). Effects of Amino Acid Supplementation to a Low-Protein Diet on the Growth Performance and Protein Metabolism-Related Factors in Broiler Chicks. J. Poult. Sci..

[14] NARO (2012). Japanese Feeding Standard for Poultry, 2011.

[15] Leary S., Pharmaceuticals F., Underwood W., Anthony R., Cartner S., Johnson C.L., Patterson-Kane E. (2020). AVMA Guidelines for the Euthanasia of Animals.

[16] Ministry of Education, Culture, Sports, Science and Technology (2015). Amino Acids. Standard Tables of Food Composition in Japan.

[17] Latimer G.W. (2023). Official Methods of Analysis of AOAC INTERNATIONAL.

[18] Suzuki T., Yasui A. (1995). Studies on the Effects of Time, Temperature and Antioxidants in the Hydrolysis Method of Food Protein for the Determination of Total Amino Acids.

[19] Bolin D.W., King R.P., Klosterman E.W. (1952). A Simplified Method for the Determination of Chromic Oxide (Cr_2_O_3_) When Used as an Index Substance. Science.

[20] Dettmer K., Stevens A.P., Fagerer S.R., Kaspar H., Oefner P.J., Alterman M.A. (2019). Amino Acid Analysis in Physiological Samples by GC-MS with Propyl Chloroformate Derivatization and ITRAQ-LC-MS/MS. Amino Acid Analysis.

[21] Thibert V. (2012). Direct Quantification of Amino Acids in Plasma by Liquid Chromatography-Tandem Mass Spectrometry for Clinical Research.

[22] Sun Z., Xing J., Khoo P.Y., Zhan Z. (2016). Direct Determination of Plasma Free Amino Acids by Combined MRM-SIM Method on LC/MS/MS.

[23] Siegert W., Omotoso A., Hofmann P., Rodehutscord M. (2025). Relevance of Nonessential Amino Acids in Low Crude Protein Diets for Broiler Chickens—An Updated Review. Nutr. Res. Rev..

[24] van Milgen J. (2021). The Role of Energy, Serine, Glycine, and 1-Carbon Units in the Cost of Nitrogen Excretion in Mammals and Birds. Animal.

[25] Cho I., An S.H., Yoon J.H., Namgung N., Kong C. (2024). Growth Performance and Nitrogen Excretion of Broiler Chickens Fed Low Protein Diets Supplemented with Crystalline Amino Acids. J. Anim. Sci. Technol..

[26] Asiamah C.A., de las Heras-Saldana S., Musigwa S., Kheravii S.K., Wu S.-B. (2025). Transcriptomic Analysis of Broiler Chickens Reveals Metabolic Adaptations to a Reduced Crude Protein Diet. Poult. Sci..

[27] Oliveira C.H., Bernardes R.D., Dias K.M.M., Ribeiro A.M., Rodrigueiro R.J.B., Koo B.K., Tak J., Park C., Calderano A.A., Albino L.F.T. (2023). Research Note: The Influence of Different Isoleucine: Lysine Ratios on the Growth Performance of Broiler Chickens Fed Low-Protein Diets. Poult. Sci..

[28] Toprak N.N., Yavaş I., Çenesiz A.A., Ceylan N., Çiftci I. (2021). Effects of Digestible Amino Acid Based Formulation of Low Protein Broiler Diets Supplemented with Valine, Isoleucine and Arginine on Performance and Protein Efficiency. Czech J. Anim. Sci..

[29] Interpretation of Avian Biochemistry—General Guidelines—Gribbles Veterinary. https://www.gribblesvets.com.au/veterinarians/our-tests/exotics-and-other/test-by-dept/biochemistry/interpretation-of-avian-biochemistry-general-guidelines/.

[30] Hermier D. (1997). Lipoprotein Metabolism and Fattening in Poultry. J. Nutr..

[31] Griffin H.D., Whitehead C.C., Broadbent L.A. (1982). The Relationship between Plasma Triglyceride Concentrations and Body Fat Content in Male and Female Broilers—A Basis for Selection?. Br. Poult. Sci..

[32] Luo C., Chen Y., Yin D., Yuan J. (2023). Effects of Different Dietary Starch Sources and Digestible Lysine Levels on Carcass Traits, Serum Metabolites, Liver Lipid and Breast Muscle Protein Metabolism in Broiler Chickens. Animals.

[33] Askri A., Rauglaudre T.D., Létourneau-Montminy M.-P., Alnahhas N. (2025). Impact of Low Crude Protein Diets Containing Animal Byproducts on Growth Performance, Nitrogen Excretion, Meat Yield, and Quality in Broiler Chickens. Can. J. Anim. Sci..

[34] Fukumoto Y., Suzuki K., Waki M., Yasuda T. (2015). Mitigation Option of Greenhouse Gas Emissions from Livestock Manure Composting. Jpn. Agric. Res. Q..

[35] Bist R.B., Subedi S., Chai L., Yang X. (2023). Ammonia Emissions, Impacts, and Mitigation Strategies for Poultry Production: A Critical Review. J. Environ. Manag..

[36] Li C., Salas W., Zhang R., Krauter C., Rotz A., Mitloehner F. (2012). Manure-DNDC: A Biogeochemical Process Model for Quantifying Greenhouse Gas and Ammonia Emissions from Livestock Manure Systems. Nutr. Cycl. Agroecosyst..

[37] Osada T., Fukumoto Y., Yamashita T., Ogino A. (2013). Manure Management for Greenhouse Gas Mitigation in Japan.

[38] Ogino A., Oishi K., Setoguchi A., Osada T. (2021). Life Cycle Assessment of Sustainable Broiler Production Systems: Effects of Low-Protein Diet and Litter Incineration. Agriculture.

